# Cost-effective soft-switching ultra-high step-up DC–DC converter with high power density for DC microgrid application

**DOI:** 10.1038/s41598-024-71436-w

**Published:** 2024-09-02

**Authors:** Ali Nadermohammadi, Pouya Abolhassani, Ali Seifi, Mohammadreza Zarrinehbafan, Pouneh Aghakhanlou, Seyed Hossein Hosseini, Mehran Sabahi

**Affiliations:** 1 Faculty of Electrical and Computer Engineering, University of Tabriz, Tabriz, 51666-16471 Iran; 2 Engineering Faculty, Near East University, 99138 Nicosia, Turkey

**Keywords:** Electrical and electronic engineering, Energy grids and networks

## Abstract

DC microgrids are integral to smart grids, enhancing grid reliability, power quality, and energy efficiency while enabling individual grid independence. They combine distributed and renewable energy sources, reducing overall energy consumption. High-gain DC–DC converters are crucial for elevating voltages from low-voltage DC sources like solar panels and wind turbines in DC microgrids. This paper introduces a non-isolated DC–DC converter designed to achieve ultra-high step-up (UHSU) voltage conversion utilizing a two-winding coupled inductor (CI). The propounded UHSU configuration achieves a substantial voltage increase by employing low duty cycles and a decreased turn ratio for the CI, resulting in a smaller core size. Moreover, this UHSU circuit incorporates soft-switching capabilities for both power switches and diodes, enhancing its efficiency. By keeping the voltage stress on the switches low, the design minimizes losses and improves overall efficiency. The operational modes are thoroughly analyzed, and comparisons with other topologies are presented to demonstrate the effectiveness of the proffered UHSU circuit. Finally, the performance of the UHSU circuit is validated through the construction and testing of a 150-W laboratory prototype operating at a switching frequency of 50 kHz, with V_in_ = 20 V and V_out_ = 300 V.

## Introduction

**Fig. 1:**
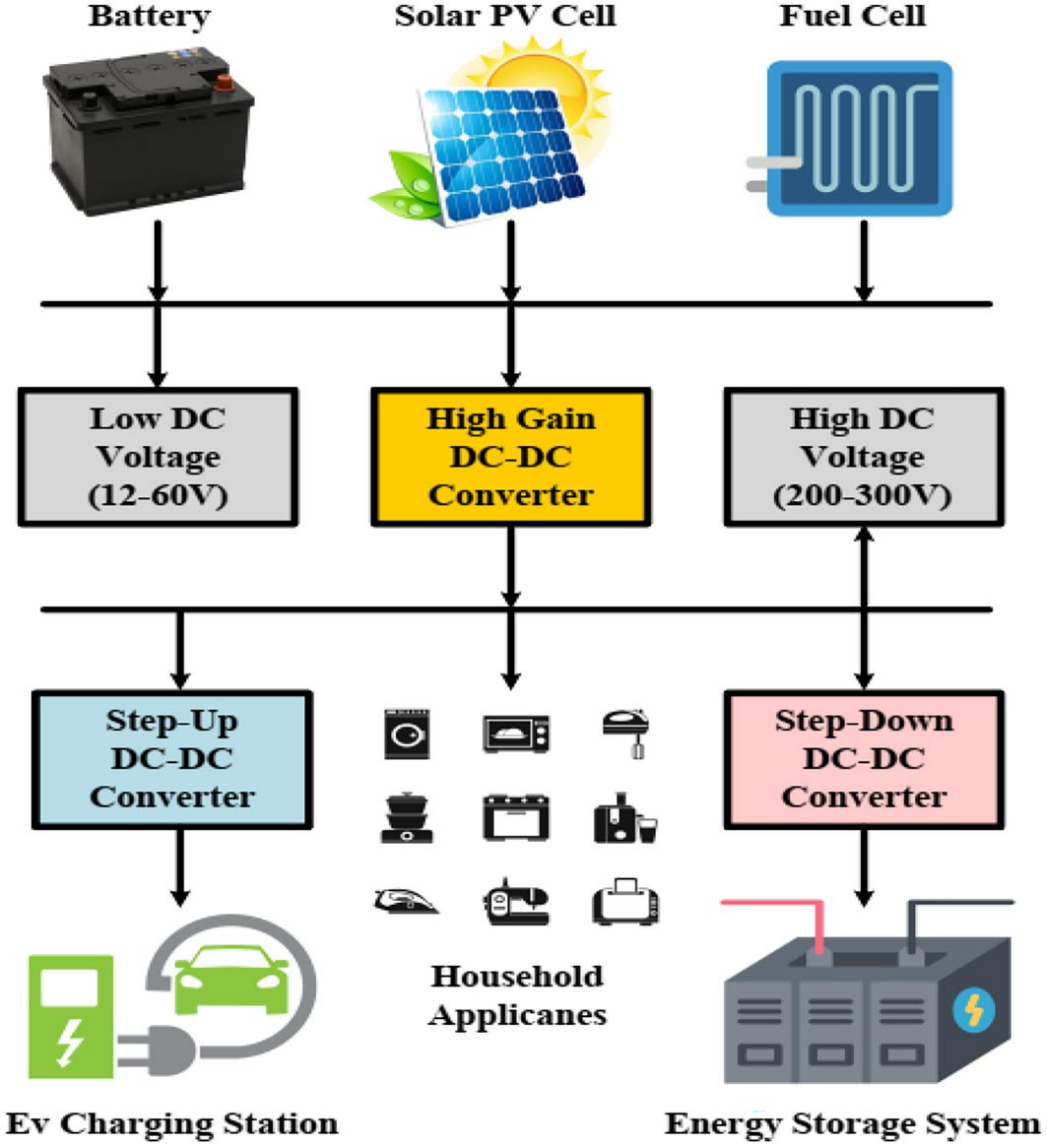
Schematic of DC microgrid.

Power electronics play a crucial role in optimizing energy extraction from renewable sources. Illustrated in Fig. 1, a DC microgrid relies on high-gain DC–DC circuits to bridge between loads and sources, elevating low voltages (12–60 V) from batteries, solar PV, and fuel cells to higher DC voltages (200–300 V). Additionally, these converters regulate the DC-link voltage to the desired level. DC–DC converters in microgrid systems exhibit a wide range of power and output voltage, divided into three main categories. Low Power and Voltage Applications span from a few watts to tens of watts with output voltages between 12 and 48 V, commonly used to power sensors, small communication devices, low-demand devices like LED lighting, and to adjust the output of small residential fuel cells (24–48 V) to align with the microgrid's voltage. Medium Power and Voltage Applications range from hundreds of watts to a few kilowatts with output voltages between 48 and 120 V, suitable for integrating medium-sized renewable energy sources, managing batteries, and supporting medium-power household devices like lighting, small appliances, and HVAC systems. High Power and Voltage Applications encompass several kilowatts to tens of kilowatts with output voltages from 120 to 400 V or more, essential for large-scale energy storage, grid-connected renewable energy sources, and power distribution within the microgrid^1–3^. In modern DC microgrids, a blend of supercapacitors and high-gain converters is used due to the supercapacitors' high power density despite their low voltage rating. Additionally, high-gain converters are crucial for level three fast charging of electric vehicles. These high step-up DC–DC circuits are applied not only in renewable energy sources like photovoltaic systems and wind turbines but also in diverse fields such as industry, physical sciences, military, medical, transportation, and aerospace, where voltage boosting is needed^4–12^. However, inherent imperfections and resistance in the step-up circuitry can lead to increased conduction losses and issues with diode reverse recovery, particularly when duty cycles are very high^4–6,13,14^.

Various methods for enhancing voltage have been explored by researchers in step-up DC–DC configurations to attain significant voltage gains without excessively large duty cycles. Comprehensive categorization of the voltage-boosting techniques is derived from five main sections: (1) multi-stage/level; (2) switched capacitor (charge pump); (3) voltage multiplier; (4) switched inductor and voltage lift; (5) magnetic coupling^15–38^.

Employing a multi-stage/level technique represents the initial approach to enhance the voltage gain of DC–DC structures, which encompasses three sections: cascade, interleave, and multilevel. However, these circuits suffer from complex control schemes, a plethora of elements, and high system costs. In recent works such as^15,39–42^, interleaved structures have been proffered, particularly suitable for photovoltaic (PV) applications due to their diminished input ripples. Switched capacitor (SC) is another prevalent voltage-boosting method based on the charge pump concept, as demonstrated in^4^, aiming to achieve high gain in DC–DC circuits. Nevertheless, SC-based circuits exhibit a significant drawback: they entail high instantaneous currents flowing through the capacitors, resulting in increased power losses and electromagnetic noise. On the other hand, the voltage multiplier technique has rendered configurations more efficient, with lower costs. These circuit topologies, relying on diodes and capacitors, are designed to achieve high voltage gain, as evidenced by works such as^43,44^. However, in studies like^45,46^, although the voltage multiplier method enables high voltage gain, the increased number of components escalates the cost and size of the circuit. Notably, the main drawback of voltage multiplier circuits lies in the high voltage stress experienced by elements. The Voltage Lift (VL) technique stands out as a widely employed method in DC–DC configurations to elevate the input voltage and attain high voltage gain^47,48^. VL involves charging a capacitor to a required voltage and leveraging it to boost the output voltage. This process can be further enhanced using additional capacitors, leading to configurations known as re-lift, triple-lift, and quadruple-lift structures. Switched inductor technique (SL), derived from both passive switched-inductor unit (PSL) and active switched-inductor unit (ASL), is another notable approach. ASL cells consist of active switches and inductors, while PSL cells comprise diodes and inductors. Studies such as^49,50^ have demonstrated that combining PSL and ASL structures can yield high voltage gain while keeping voltage stress across the power switches low. However, VL and SL techniques require additional passive elements and may not be appropriate for high-power applications. The magnetic coupling technique emerges as a promising solution for high step-up circuits. By utilizing CI in configurations, the number of components can be diminished compared to discrete inductors. CI-based converters offer an additional degree of freedom owing to their turn ratio, which can significantly increase voltage gain. Works such as^15,16,24,26,27,51^ have shown that adjusting the turns ratio among the primary, secondary, and tertiary windings of the CI can lead to remarkably high voltage gain in configurations.

This study presents a non-isolated DC–DC structure aimed at achieving ultra-high step-up voltage conversion through the use of a CI method. By adopting this approach, the voltage stress on the power switches is effectively diminished, allowing for the employment of low-voltage-rated switches with minimal on-resistance. As a result, conduction losses are minimized, ultimately contributing to improved efficiency.

The salient benefits of the propounded configuration are as:

1. To manage the voltage gain of the configuration, the proffered design offers two adjustable parameters: the duty cycle of the power switches and the turns ratio of the CIs. This flexibility in design allows for effective control over the converter's voltage gain.
2. The voltage stress on the power switches is minimal, allowing for the use of low-voltage-rated switches with decreased on-resistance. This choice helps to mitigate conduction losses and ultimately enhances the total efficiency of the system.
3. The proffered configuration can produce a substantial output voltage with a minimal duty cycle, thereby decreasing the conduction loss of the switches.
4. Utilizing one core in the propounded structure reduces the size and cost of the circuit and enhances the power density.
5. Both power switches and three of the diodes have zero current switching (ZCS), which leads to enhanced efficiency.

## Proposed converter and operation modes

**Fig. 2:**
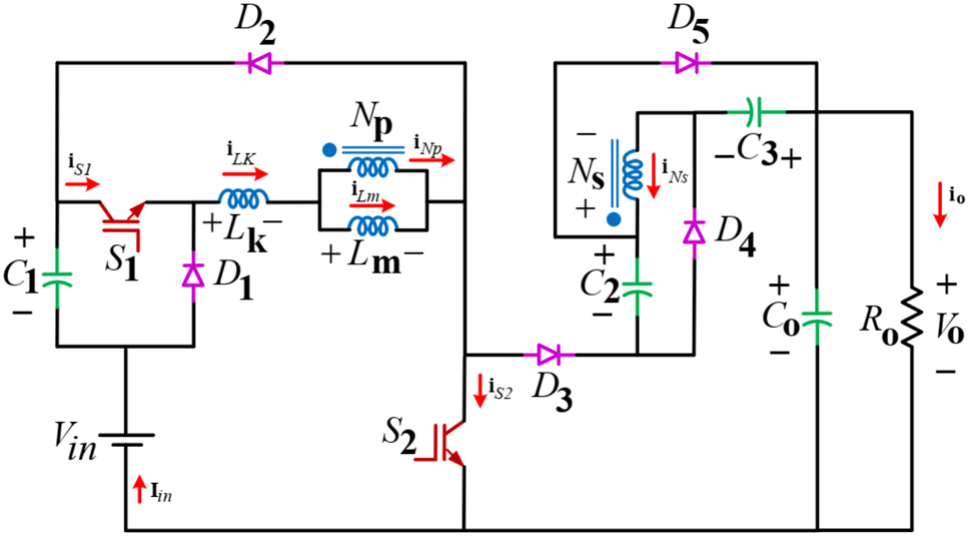
Power circuit of the propounded configuration.

**Fig. 3:**
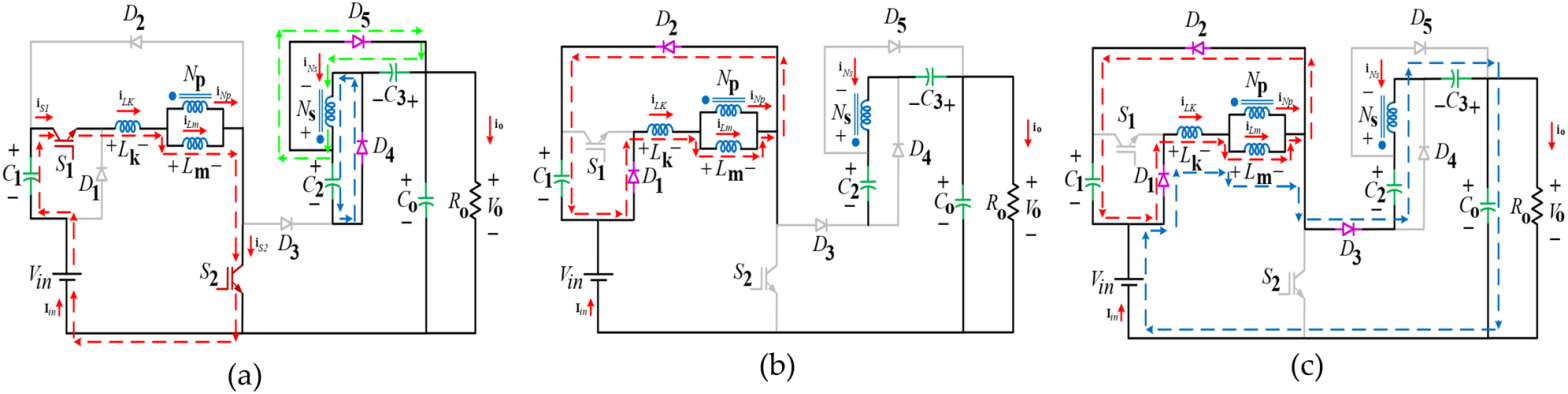
Equivalent circuits of the proffered circuit in boost mode (**a**) First switching subinterval, (**b**) Second switching subinterval, (**c**) Third switching subinterval.

**Fig. 4:**
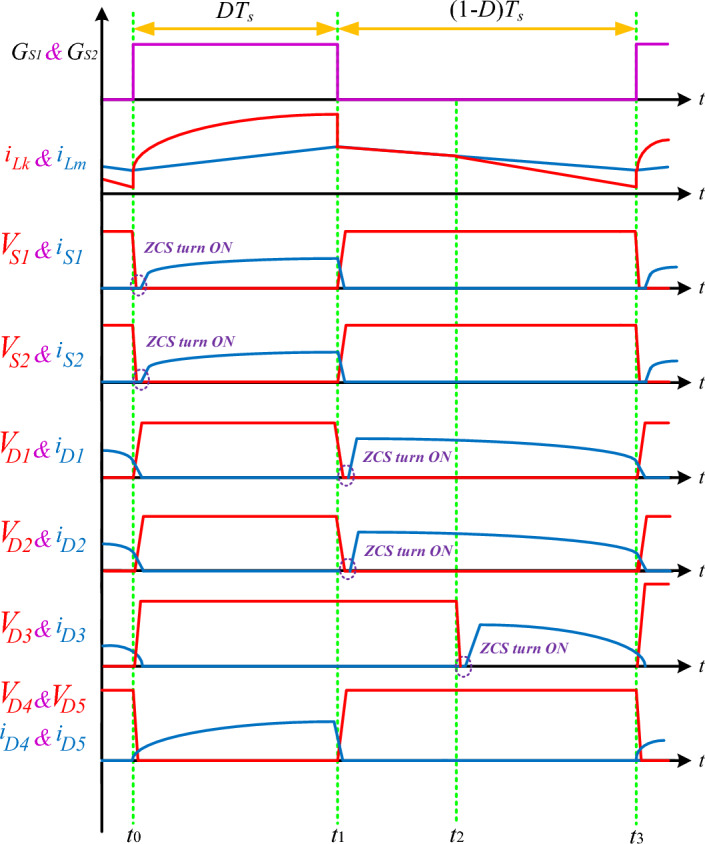
Main waveforms of the proffered circuit.

The propounded configuration depicted in Fig. 2 illustrates the design of the presented configuration. This configuration includes two switches (S_1_ and S_2_), four capacitors (C_1_–C_3_ and C_O_), five diodes (D_1_–D_5_), and a coupled inductor with a two-winding configuration featuring a primary side winding (N_P_) and a secondary side winding (N_S_). Furthermore, the turns ratio of this coupled inductor is expressed as n = N_S_/N_P_, and the coupling coefficient is represented as k = L_m_/(L_m_ + L_k_). The propounded UHSU circuit, operating in continuous conduction mode (CCM), can be segmented into three subintervals, as demonstrated in Fig. 3. The primary waveforms of voltage and current for these components are illustrated in Fig. 4.

### First switching subinterval

In this operational mode, both power switches are activated in zero-current-switching, while diodes D_4_ and D_5_ are forward-biased, and the remaining diodes are in a reverse-biased state. The magnetizing inductor L_m_ charges through the circuit path including V_in_, C_1_, S_1_, L_m_, and S_2_, resulting in a linear increase in i_Lm_. At the same time, capacitor C_2_ charges via the loop consisting of C_2_, D_4_, and N_S_, and capacitor C_3_ charges through the circuit path comprising C_3_, N_S_, and D_5_. Throughout this time interval, the following relationships are maintained according to Kirchhoff's Voltage Law (KVL).

1 $$V_{Ns} = nV_{Lm}$$

2 $$V_{1} = V_{Lm} + V_{Lk} = \frac{{V_{Lm} }}{k}$$

3 $$V_{in} = - V_{C1} + V_{1}$$

4 $$V_{Ns} = V_{C2}$$

5 $$V_{C3} = V_{Ns}$$

### Second switching subinterval

In this interval, both power switches are turned off, and diodes D_1_ and D_2_ function under forward-biased conditions with ZCS conditions, while the remaining diodes are off. The magnetizing inductor's current decreases linearly. Capacitor C_1_ charges through the circuit loop involving C_1_, D_1_, L_m_, and D_2_. According to KVL, the relationship governing this mode can be articulated as follows.

6 $$V_{1} = - V_{C1}$$

### Third switching subinterval

In this phase of operation, both power switches remain inactive, and diodes D_1_ and D_2_ continue conducting in a forward-biased state. Diode D_3_ enters conduction with ZCS conditions, while diodes D_4_ and D_5_ are in a reverse-biased state. The current in inductor L_m_ decreases steadily. Capacitor C_1_ charges through the circuit loop comprising C_1_, D_1_, L_m_, and D_2_. Simultaneously, the output voltage V_O_ is charged through the circuit path involving V_in_, D_1_, L_m_, D_3_, C_2_, N_S_, C_3_, and V_O_. According to KVL, the relationships governing this mode can be articulated as follows.

7 $$V_{in} + V_{C1} + V_{C2} - V_{Ns} + V_{C3} = V_{O}$$

8 $$- V_{1} = V_{C1}$$

9 $$V_{O} = - V_{1} + V_{in} + V_{C2} + V_{C3} - V_{Ns}$$

### Voltage gain calculation

The principle of volt-second equilibrium for the CI can be applied in the following way:

10 $$\left\langle {V_{Lm} } \right\rangle_{{T_{S} }} = 0$$

11 $$\left\langle {V_{1} } \right\rangle_{{T_{S} }} = 0$$

The capacitor voltages, obtained from Eqs. (12) and (13), are outlined below:

12 $$V_{C1} = \frac{{DV_{in} }}{1 - 2D}$$

13 $$V_{C2} = V_{C3} = \frac{{V_{in} kn(1 - D)}}{1 - 2D}$$

The formulated representation for the output voltage of the given setup is as follows:

14 $$V_{O} = \frac{{V_{in} (1 + 2kn - D - nkD)}}{1 - 2D}$$

Neglecting the CI coefficient (especially for k = 1), the gain in output voltage should be expressed as:

15 $$G = \frac{{V_{O} }}{{V_{in} }} = \frac{1 + 2n - D - nD}{{1 - 2D}}$$

### Voltage stresses of semiconductors

The voltage across the circuit switches and diodes in the setup can be delineated as follows:

16 $$V_{S1} = \frac{{DV_{in} }}{1 - 2D}$$

17 $$V_{S2} = \frac{{(1 - D)V_{in} }}{1 - 2D}$$

18 $$V_{D1} = \frac{{DV_{in} }}{1 - 2D}$$

19 $$V_{D2} = \frac{{(1 - D)V_{in} }}{1 - 2D}$$

20 $$V_{D3} = \frac{{(kn - D + 1)V_{in} }}{1 - 2D}$$

21 $$V_{D4} = V_{D5} = \frac{{knV_{in} }}{1 - 2D}$$

### Average currents of semiconductors

The average currents flowing through the power switches and diodes can be described as follows:

22 $$I_{S1} = I_{S2} = \frac{{(D + 2n - Dn)I_{O} }}{1 - 2D}$$

23 $$I_{D1} = \frac{{(1 - D + 2n - Dn)I_{O} }}{1 - 2D}$$

24 $$I_{D2} = \frac{{(D + 2n - Dn)I_{O} }}{1 - 2D}$$

25 $$I_{D3} = I_{D4} = I_{D5} = I_{O}$$

### Boundary condition

For the CCM operation of the suggested circuit, the minimum current of the coupled inductor must be more than zero. The minimum current of the CI and $\Delta_{iLm}$ can be calculated as:

26 $$I_{Lm} = \frac{{(3n + 1)I_{O} }}{1 - 2D}$$

27 $$\Delta I_{Lm} = \frac{{D \times V_{in} \times \left( {1 + \frac{D}{1 - 2D}} \right)}}{{L_{m} f_{s} }}$$

28 $$\left\{ \begin{gathered} I_{Lm,\min } = I_{Lm} - \frac{{\Delta I_{Lm} }}{2} \hfill \\ I_{Lm,\max } = I_{Lm} + \frac{{\Delta I_{Lm} }}{2} \hfill \\ \end{gathered} \right.$$

29 $$L_{m} \ge \frac{D \times (1 - 2D + D) \times (1 - 2D) \times R}{{2 \times (3n + 1) \times (1 + 2n - D - Dn) \times f_{s} }}$$

Normalized CI time constant can be expressed as:

30 $$\tau = \frac{{2L_{m} f_{s} }}{R}$$

The boundary-normalized CI time constant can be described by employing (26), (27), (28) and (29).

31 $$\tau_{B} = \frac{D \times (1 - 2D + D) \times (1 - 2D)}{{(3n + 1) \times (1 + 2n - D - Dn)}}$$

During continuous conduction mode, $\tau$ needs to surpass $\tau_{B}$. Conversely, in discontinuous conduction mode (DCM), $\tau_{B}$ exceeds $\tau$, while in boundary conduction mode (BCM), $\tau$ and $\tau_{B}$ must maintain equality.

32 $$\left\{ \begin{gathered} \tau > \tau_{B} ;CCM \hfill \\ \tau < \tau_{B} ;DCM \hfill \\ \tau = \tau_{B} ;BCM \hfill \\ \end{gathered} \right.$$

**Fig. 5:**
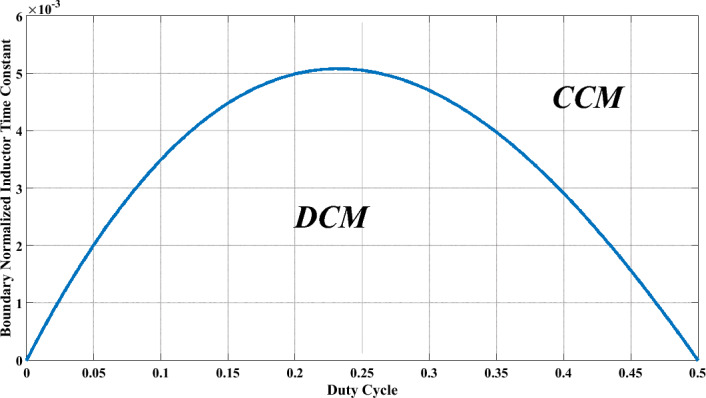
Boundary normalized inductor time constant versus duty cycle.

The depicted converter has the capability to function across different zones, contingent upon Eq. (32), as showcased in Fig. 5.

### Efficiency calculation

In this section, calculations are performed to assess the conduction and switching losses of the proffered structure with the objective of optimizing its efficiency. To achieve this, factors such as the internal resistances of diodes (r_D_), switches (r_S_), inductors (r_Lm_), capacitors (r_C_), as well as the forward voltage drop across diodes (V_FD_) and switches (V_FS_) are taken into account in determining power losses. Consequently, the conduction losses of switches and diodes are derived as follows:

33 $$P_{{Cond,S_{1} }} = \frac{{Dr_{s} {\mkern 1mu} {\text{I}}_{O}^{2} \left( {D + 2n - Dn} \right)^{2} }}{{\left( {2D - 1} \right)^{2} }} - \frac{{D{\mkern 1mu} {\text{V}}_{{{\text{Fs}}}} {\mkern 1mu} {\text{I}}_{O} \left( {D + 2n - Dn} \right)}}{2D - 1}$$

34 $$P_{{Cond,S_{2} }} = \frac{{Dr_{s} {\mkern 1mu} {\text{I}}_{O}^{2} \left( {D + 2n - Dn} \right)^{2} }}{{\left( {2D - 1} \right)^{2} }} - \frac{{D{\mkern 1mu} {\text{V}}_{{{\text{Fs}}}} {\mkern 1mu} {\text{I}}_{O} \left( {D + 2n - Dn} \right)}}{2D - 1}$$

35 $$\left\{ \begin{gathered} P_{{Cond,D_{1} }} = - \frac{{r_{{D_{1} }} {\mkern 1mu} {\text{I}}_{O}^{2} (D - 1)\left( {D - 2n + Dn - 1} \right)^{2} }}{{\left( {2D - 1} \right)^{2} }} \hfill \\ - \frac{{{\mkern 1mu} {\text{V}}_{{{\text{FD}}_{{1}} }} {\mkern 1mu} {\text{I}}_{O} (D - 1)\left( {D - 2n + Dn - 1} \right)}}{2D - 1} \hfill \\ \end{gathered} \right.$$

36 $$\left\{ \begin{gathered} P_{{Cond,D_{2} }} = - \frac{{r_{{D_{2} }} {\mkern 1mu} {\text{I}}_{O}^{2} (D - 1)\left( {D - 2n + Dn} \right)^{2} }}{{\left( {2D - 1} \right)^{2} }} \hfill \\ - \frac{{{\mkern 1mu} {\text{V}}_{{{\text{FD}}_{{2}} }} {\mkern 1mu} {\text{I}}_{O} (D - 1)\left( {D - 2n + Dn} \right)}}{2D - 1} \hfill \\ \end{gathered} \right.$$

37 $$P_{{Cond,D_{3} }} = - \frac{{{\text{3I}}_{o} {\mkern 1mu} \left( {D - 1} \right){\mkern 1mu} \left( {{\text{V}}_{{{\text{FD}}_{3} }} + {\text{I}}_{O} {\mkern 1mu} {\text{r}}_{{{\text{D}}_{3} }} } \right)}}{5}$$

38 $$P_{{Cond,D_{4} }} = D{\text{I}}_{o} ({\mkern 1mu} {\text{r}}_{{{\text{D}}_{4} }} {\mkern 1mu} {\text{I}}_{{\text{o}}} + {\mkern 1mu} {\text{V}}_{{{\text{FD}}_{4} }} {\mkern 1mu} )$$

39 $$P_{{Cond,D_{5} }} = D{\text{I}}_{o} ({\mkern 1mu} {\text{r}}_{{{\text{D}}_{5} }} {\mkern 1mu} {\text{I}}_{{\text{o}}} + {\mkern 1mu} {\text{V}}_{{{\text{FD}}_{5} }} {\mkern 1mu} )$$

The computation of switching losses for the power switches and diodes is carried out as follows:

40 $$P_{{SW,S_{1} }} = \frac{{D \times {\text{I}}_{O} {\mkern 1mu} \times {\text{V}}_{{{\text{in}}}} \times f_{s} \times t_{off} \times (D + 2n - Dn)}}{{6 \times (2D - 1)^{2} }}$$

41 $$P_{{SW,S_{2} }} = - \frac{{{\text{I}}_{O} {\mkern 1mu} \times {\text{V}}_{{{\text{in}}}} \times f_{s} \times t_{off} \times (D - 1) \times (D + 2n - Dn)}}{{6 \times (2D - 1)^{2} }}$$

42 $$P_{{SW,D_{1} }} = - \frac{{D \times I_{rr} \times V_{in} \times f_{s} \times t_{b} }}{6 \times (2D - 1)}$$

43 $$P_{{SW,D_{2} }} = \frac{{(D - 1) \times I_{rr} \times V_{in} \times f_{s} \times t_{b} }}{6 \times (2D - 1)}$$

44 $$P_{{SW,D_{3} }} = - \frac{{(n - D + 1) \times I_{rr} \times V_{in} \times f_{s} \times t_{b} }}{6 \times (2D - 1)}$$

45 $$P_{{SW,D_{4} }} = - \frac{{n \times I_{rr} \times V_{in} \times f_{s} \times t_{b} }}{6 \times (2D - 1)}$$

46 $$P_{{SW,D_{5} }} = - \frac{{n \times I_{rr} \times V_{in} \times f_{s} \times t_{b} }}{6 \times (2D - 1)}$$

The overall power loss of the power switches and diodes, encompassing both switching and conduction losses, is represented as follows:

47 $$P_{S,Tot} = P_{{Cond,S_{1} }} + P_{{SW,S_{1} }} + P_{{Cond,S_{2} }} + P_{{SW,S_{2} }}$$

48 $$P_{D,Tot} = P_{{Cond,D_{1,2,3,4,5} }} + P_{{SW,D_{1,2,3,4,5} }}$$

The conduction losses associated with capacitors C_1_, C_2_, C_3_, and C_O_ are determined as follows:

49 $$\left\{ \begin{gathered} P_{{Cond,C_{1} }} = \frac{{r_{{C_{1} }} {\mkern 1mu} {\text{I}}_{O}^{2} D\left( {D + 2n - Dn} \right)^{2} }}{{\left( {2D - 1} \right)^{2} }} \hfill \\ - \frac{{{\mkern 1mu} r_{{C_{1} }} {\mkern 1mu} {\text{I}}_{O}^{2} (D - 1)\left( {D - 2n + Dn} \right)^{2} }}{{\left( {2D - 1} \right)^{2} }} \hfill \\ \end{gathered} \right.$$

50 $$P_{{Cond,C_{2} }} = \frac{{r_{{C_{2} }} {\text{I}}_{o}^{2} ({\mkern 1mu} 2D + 3{\mkern 1mu} )}}{5}$$

51 $$P_{{Cond,C_{3} }} = \frac{{r_{{C_{3} }} {\text{I}}_{o}^{2} ({\mkern 1mu} 2D + 3{\mkern 1mu} )}}{5}$$

52 $$\left\{ \begin{gathered} P_{{Cond,C_{O} }} = \frac{{12r_{{C_{O} }} {\mkern 1mu} {\text{I}}_{O}^{2} }}{5} - \frac{{7r_{{C_{O} }} {\mkern 1mu} {\text{I}}_{O}^{2} D}}{5} \hfill \\ - \frac{{{\mkern 1mu} 2r_{{C_{O} }} {\mkern 1mu} {\text{I}}_{O}^{2} (D - 1)\left( {D - 2n + Dn} \right)^{2} }}{{5\left( {2D - 1} \right)^{2} }} \hfill \\ \end{gathered} \right.$$

The overall power loss of the capacitors is represented as follows:

53 $$P_{C,Tot} = P_{{Cond,C_{1} }} + P_{{Cond,C_{2} }} + P_{{Cond,C_{3} }} + P_{{Cond,C_{O} }}$$

The expression for the conduction loss of the magnetizing inductor L_m_ is formulated as follows:

54 $$P_{Cond,Lm} = r_{Lm} I_{Lm}^{2} = \frac{{{\text{Io}}^{2} r_{Lm} (3n + 1)^{2} }}{{(2D - 1)^{2} }}$$

The power loss associated with the core of the CI is denoted as follows:

55 $$P_{C} = kf_{s}^{\alpha } B_{m}^{\beta }$$

The power loss of the coupled inductor core (PC) is measured in W/kg. The coefficients utilized for the core, namely α, β, and k, are known as Steinmetz parameters, and they are often provided by producers for different core materials. The value of the α coefficient can vary between 1 and 2 for ferrite materials (1 ≤ α ≤ 2). based on Faraday's Law, this can be expressed as:

56 $$V_{L} = N\frac{d\varphi (t)}{{dt}} = NA_{c} \frac{dB(t)}{{dt}}$$

The core area, denoted as A_c_, is specified by manufacturers for different types of magnetic cores. N represents the turn ratio of the CI. Consequently, the peak flux density, denoted as ΔB, for the CI can be determined as follows:

57 $$\Delta B = \frac{1}{{NA_{c} }}\int\limits_{0}^{{DT_{s} }} {V_{in} dt = } \frac{{V_{in} DT_{s} }}{{N_{P} A_{c} }} = \frac{{V_{in} D}}{{N_{P} A_{c} f_{s} }}$$

The core loss for the CI is represented as P_Core_ = P_C_M, where M denotes the mass of the coupled inductor core. Hence, with B_m_ = ΔB/2 taken into account, the core loss is formulated as follows:

58 $$P_{Core} = kf_{s}^{\alpha } \left( {\frac{{V_{in} D}}{{2N_{P} A_{c} f_{s} }}} \right)^{\beta } M$$

Referencing the book "Transformer and Inductor Design Handbook" authored by Colonel Wm. T. McLyman, the parameters employed to compute core losses include k = 5.597 × 10 − 4, α = 1.43, β = 2.85, B_m_ = 0.1 T, M = 0.088 kg, and f_s_ = 50 kHz.

Therefore, the total power losses for the ultra-high step-up configuration are computed as follows:

59 $$P_{Loss} = P_{S,Tot} + P_{D,Tot} + P_{Cond,Lm} + P_{Core} + P_{C,Tot}$$

The efficiency of the proffered circuit (*η*) is determined as:

60 $$\eta = \frac{{P_{Out} }}{{P_{Out} + P_{Loss} }}$$

where P_Out_ represents the output power of the proposed configuration, defined as P_Out_ = V_O2_/R_O_.

**Fig. 6:**
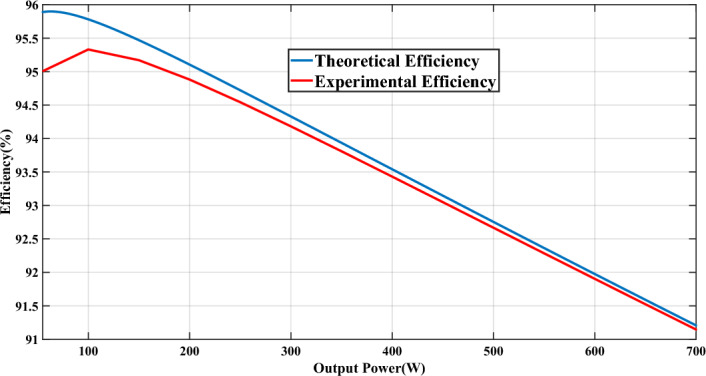
The theoretical and experimental efficiency of the proffered step-up configuration versus output power.

**Fig. 7:**
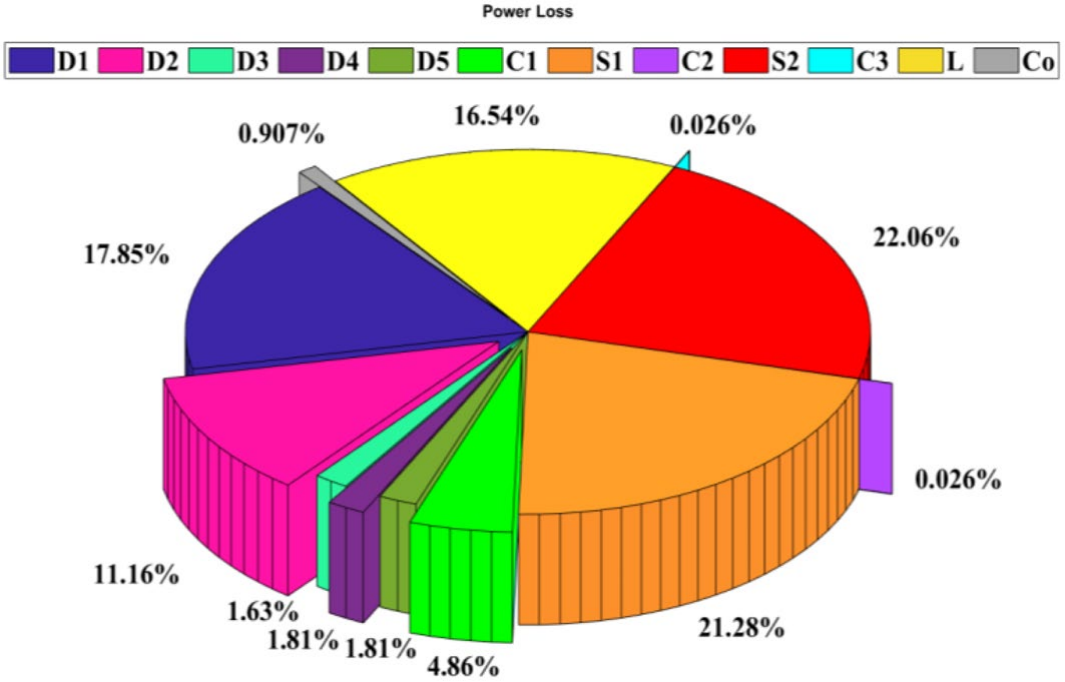
Calculated power loss percentages for the components (P_O_ = 150W, V_in_ = 20 V and Vo = 300 V).

**Fig. 8:**
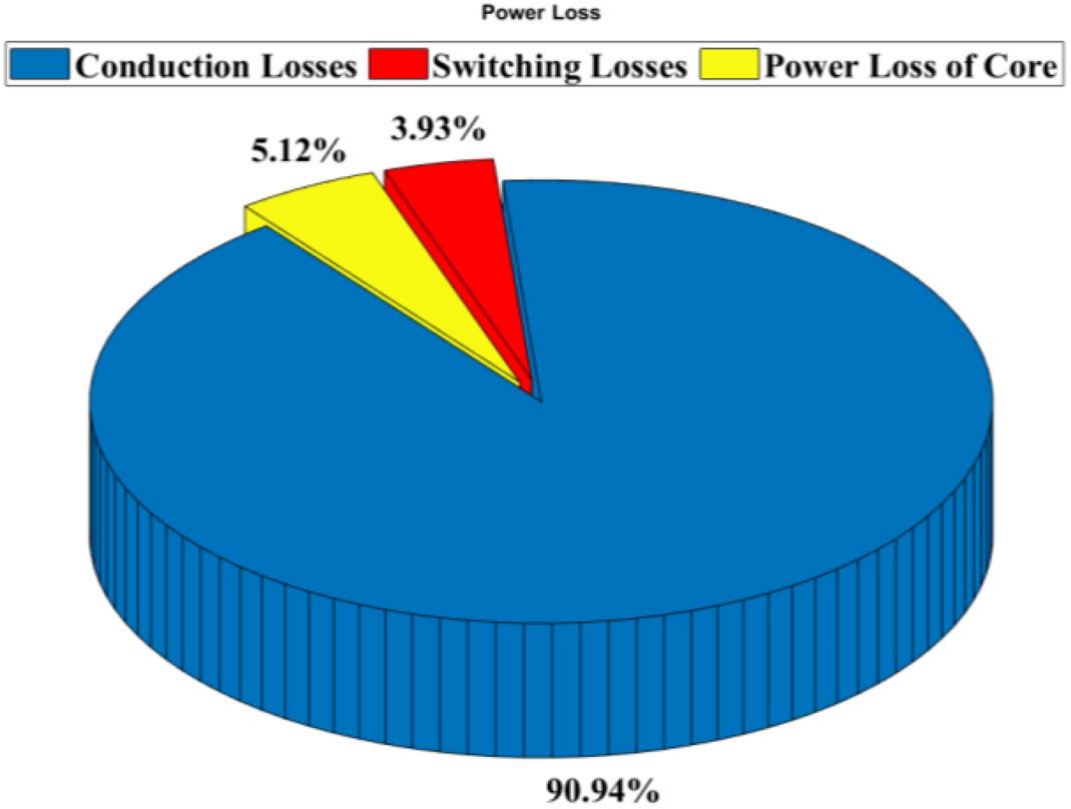
Calculated power loss percentages for conduction losses, switching losses, and core loss of the coupled inductor (P_O_ = 150W, V_in_ = 20 V and Vo = 300 V).

By referencing Eqs. (33) to (60), the analytical efficiency of the propounded circuit can be derived, and the theoretical and experimental efficiencies of the proffered circuit versus output power are graphed in Fig. 6. Figure 7 illustrates the percentage distribution of power loss for each component type, while Fig. 8 provides a breakdown of power loss percentages specifically for conduction losses, switching losses, and core loss of the CI. As seen in Fig. 7, approximately half of the total power loss is attributed to the power switches, mainly due to their conduction losses. In Fig. 8, the switching losses of the diodes and switches, resulting from ZCS condition, are comparatively lower than both the conduction losses and the power loss of the CI core.

### Key parameter design guidance

#### Capacitors voltage ripple in boost mode

The capacitors' design characteristics are determined by averaging capacitor currents across all switching subintervals, considering capacitor voltages, duty cycles, allowable fluctuation range _C_%, and a prescribed switching frequency of 50 kHz. Consequently, the minimum values for capacitors C_1_ to C_O_ can be computed as follows:

61 $$C_{1} \ge \frac{{I_{O} \times (2D - D - Dn) \times (1 - D)}}{{f_{s} \times V_{in} \times D \times x_{C1} \% }}$$

62 $$C_{2} \ge \frac{{I_{O} \times D \times (1 - 2D)}}{{f_{s} \times V_{in} \times n(1 - D) \times x_{C2} \% }}$$

63 $$C_{3} \ge \frac{{I_{O} \times D \times (1 - 2D)}}{{f_{s} \times V_{in} \times n(1 - D) \times x_{C3} \% }}$$

64 $$C_{O} \ge \frac{{2I_{O} \times (1 - D) \times (1 - 2D)}}{{f_{s} \times V_{in} (1 + 2n - D - nD) \times x_{Co} \% }}$$

### Coupled inductor design

The design specifics of the CI rely on several factors, including the average currents flowing through it, the voltage applied across the CI throughout all switching intervals, the duty cycle, the allowable fluctuation range denoted as _L_%, and the frequency of switching. Consequently, the minimum value required for the magnetizing inductor can be formulated as follows:

65 $$L_{m} \ge \frac{{V_{in} \times (D - 2D^{2} + D^{2} )}}{{f_{s} \times {\text{Io}}{\mkern 1mu} \left( {3{\mkern 1mu} n + 1} \right) \times x_{Lm} \% }}$$

### Number of primary and secondary winding turns of coupled inductor

The selected magnitude for the leakage inductor (L_k_) is 3 µH. Consequently, the determination of the magnetizing inductor's value can be expressed utilizing the coupling coefficient of the CI as follows:

66 $$L_{m} = \frac{{3k \times 10^{ - 6} }}{1 - k}$$

The magnetic core E42/21/15 has been selected for the CI. Consequently, according to the dimensions provided in the core datasheet, the air gaps of the core for the CI can be defined as follows:

67 $$l_{g} = \frac{{L_{m} I_{Lm}^{2} \mu_{0} }}{{B_{m}^{2} A_{{Air{\text{ Gap}}}} }} = \frac{{\frac{{3k \times 10^{ - 6} }}{1 - k} \times \left( {\left. {\frac{{{\text{Io}}{\mkern 1mu} \left( {3{\mkern 1mu} n + 1} \right)}}{1 - 2D}} \right)} \right.^{2} \times 4\pi \times 10^{ - 7} }}{{0.1^{2} \times [(12.2 + 12.5) \times 15.2 \times 10^{ - 6} ]}}$$

Through the utilization of Eq. (61), the count of primary winding turns for the CI can be obtained using the subsequent formula:

68 $$N_{P} \ge \sqrt {L_{m} \times \frac{{l_{g} }}{{\mu_{0} A_{{Air{\text{ Gap}}}}^{Equivalent} }}}$$

The ratio of turns for the CI, denoted as n = N_S_/N_P_, establishes the count of secondary winding turns, which can be articulated as follows:

69 $$N_{S} = n \times N_{P} \Rightarrow N_{S} = 1.5 \times N_{P}$$

## Small-signal modeling

Each power semiconductor, the coupled inductor, and the capacitors are considered ideal in this analysis. The CI includes parasitic series resistors denoted as r_Lm_, while the parasitic series resistors of capacitors are labeled as r_C_. Utilizing the state-space averaging method enables the derivation of both the average model and the small-signal model. System equations are formulated for all modes, and they are averaged over each commutation time, accounting for the time interval of each mode. Throughout all three switching subintervals, the system equations are expressed as follows:

70 $$\left[ {\begin{array}{*{20}c} {\dot{I}_{Lm} } \\ {\dot{V}_{C1} } \\ {\dot{V}_{C2} } \\ {\dot{V}_{C3} } \\ {\dot{V}_{CO} } \\ \end{array} } \right] = [A_{m} ]\left[ {\begin{array}{*{20}c} {I_{Lm} } \\ {V_{C1} } \\ {V_{C2} } \\ {V_{C3} } \\ {V_{CO} } \\ \end{array} } \right] + [B_{m} ]V_{in}$$

where m = 1, 2, 3.

The control strategy for the presented converter employs the pole placement method, and the small signal model of the circuit is deduced from the state-space averaged model. Through the small signal modeling approach, state variables and control inputs are delineated into two components: fixed ($\overline{X},\overline{D}$) and variable ($\tilde{x},\tilde{d}$).

71 $$\left\{ \begin{gathered} X = \overline{X} + \tilde{x} \hfill \\ D = \overline{D} + \tilde{d} \hfill \\ \end{gathered} \right.$$

Applying this to the average model of the state space and disregarding their squared values, we derive the small signal model of the proffered structure.

72 $$\left\{ \begin{gathered} \dot{\tilde{x}} = A\tilde{x} + B\tilde{u} \hfill \\ y = C\tilde{x} + D\tilde{u} \hfill \\ \end{gathered} \right.$$

where variable states ($\tilde{x}$), control inputs ($\tilde{u}$), and output signals (*y*) are described as follows:

73 $$\tilde{x}^{T} = \left[ {\begin{array}{*{20}c} {\tilde{i}_{Lm} } & {\tilde{v}_{C1} } & {\tilde{v}_{C2} } & {\tilde{v}_{C3} } & {\tilde{v}_{CO} } \\ \end{array} } \right]$$

74 $$\tilde{u} = \left[ {\tilde{d}} \right]$$

75 $$y^{T} = \left[ {I_{Lm} } \right]$$

According to the pole placement technique, the poles of the closed loop can be positioned at any suitable location given that the system is fully state-controllable. The controllability matrix of the proffered circuit is articulated as follows:

76 $$\Phi_{C} = \left[ {B \,\,\vdots \,\,AB \,\,\vdots \,\,A^{2} \,\,B \,\,\vdots \,\cdots \,\vdots \,\,A^{n - 1} B} \right]$$

If the rank of $\Phi_{C}$ equals 5, which is equivalent to the number of variable states ($\tilde{x}$), then the system is considered fully controllable. Subsequently, two additional integral states are derived as follows:

77 $$\dot{q}(t) = r(t) - y(t) = r(t) - \tilde{i}_{Lm} (t)$$

With the inclusion of the new integral states, the state and output equations are reformulated as follows:

78 $$\begin{aligned} \left[ {\begin{array}{*{20}c} {\dot{\tilde{x}}(t)} \\ \cdots \\ {\dot{q}(t)} \\ \end{array} } \right] & = \left[ {\begin{array}{*{20}c} A & \vdots & 0 \\ \cdots & \vdots & \cdots \\ { - C} & \vdots & 0 \\ \end{array} } \right]\left[ {\begin{array}{*{20}c} {\tilde{x}(t)} \\ \cdots \\ {q(t)} \\ \end{array} } \right] + \left[ {\begin{array}{*{20}c} B \\ \cdots \\ 0 \\ \end{array} } \right]\tilde{u}(t) + \left[ {\begin{array}{*{20}c} 0 \\ \cdots \\ I \\ \end{array} } \right]r(t) \\ y(t) & = \left[ {\begin{array}{*{20}c} C & \vdots & 0 \\ \end{array} } \right]\left[ {\begin{array}{*{20}c} {\tilde{x}(t)} \\ \cdots \\ {q(t)} \\ \end{array} } \right] \\ \end{aligned}$$

In the equation provided, r(t) represents the input reference vector, defined as follows:

79 $$r(t) = \left[ {I_{Lm,ref} } \right]^{T}$$

According to Eq. (78), the new matrixes $\overline{A}$ and $\overline{B}$ are articulated as follows:

80 $$\overline{A} = \left[ {\begin{array}{*{20}c} A & \vdots & 0 \\ \cdots & \vdots & \cdots \\ { - C} & \vdots & 0 \\ \end{array} } \right],\overline{B} = \left[ {\begin{array}{*{20}c} B \\ \cdots \\ 0 \\ \end{array} } \right]$$

The controllability matrix for the system described in Eq. (78) can be defined as follows:

81 $$\overline{\Phi }_{C} = \left[ {\begin{array}{*{20}c} B & \vdots & {A\Phi_{C} } \\ \cdots & \vdots & \cdots \\ 0 & \vdots & { - C\Phi_{C} } \\ \end{array} } \right] = \underbrace {{\left[ {\begin{array}{*{20}c} B & \vdots & A \\ \cdots & \vdots & \cdots \\ 0 & \vdots & { - C} \\ \end{array} } \right]}}_{M}\left[ {\begin{array}{*{20}c} I & \vdots & 0 \\ \cdots & \vdots & \cdots \\ 0 & \vdots & {\Phi_{C} } \\ \end{array} } \right]$$

If $\Phi_{C}$ is established to be complete-rank, the system mentioned in Eq. (78) is entirely controllable if the rank of the matrix M is n + m (where n and m represent the number of variable states ($\tilde{x}$) and output signals (y), respectively). Therefore, there exists a matrix K calculated as:

82 $$\tilde{u}(t) = - K\left[ {\begin{array}{*{20}c} {\tilde{x}(t)} \\ \cdots \\ {q(t)} \\ \end{array} } \right] = - \left[ {\begin{array}{*{20}c} {K_{x} } & \vdots & {K_{q} } \\ \end{array} } \right]\left[ {\begin{array}{*{20}c} {\tilde{x}(t)} \\ \cdots \\ {q(t)} \\ \end{array} } \right]$$

where K_x_ and K_q_ expressed as follows:

83 $$\begin{gathered} K_{x} = \left[ {\begin{array}{*{20}c} {K_{11} } & {K_{12} } & {K_{13} } & {K_{14} } & {K_{15} } \\ \end{array} } \right] \hfill \\ K_{q} = \left[ {K^{\prime}_{11} } \right] \hfill \\ \end{gathered}$$

Substituting (82) in (78) the following equation is:

84 $$\begin{aligned} \left[ {\begin{array}{*{20}c} {\dot{\tilde{x}}(t)} \\ \cdots \\ {\dot{q}(t)} \\ \end{array} } \right] & = \left[ {\begin{array}{*{20}c} {A - BK_{x} } & \vdots & { - BK_{q} } \\ \cdots & \vdots & \cdots \\ { - C} & \vdots & 0 \\ \end{array} } \right]\left[ {\begin{array}{*{20}c} {\tilde{x}(t)} \\ \cdots \\ {q(t)} \\ \end{array} } \right] + \left[ {\begin{array}{*{20}c} 0 \\ \cdots \\ I \\ \end{array} } \right]r(t) \\ y(t) & = \left[ {\begin{array}{*{20}c} C & \vdots & 0 \\ \end{array} } \right]\left[ {\begin{array}{*{20}c} {\tilde{x}(t)} \\ \cdots \\ {q(t)} \\ \end{array} } \right] \\ \end{aligned}$$

**Fig. 9:**
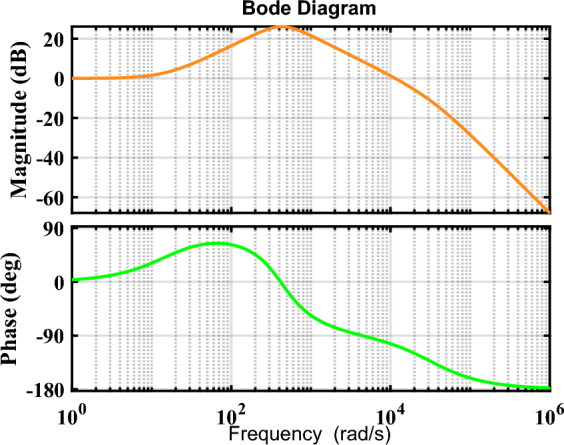
Bode diagram for the transfer function of the inductor current.

**Fig. 10:**
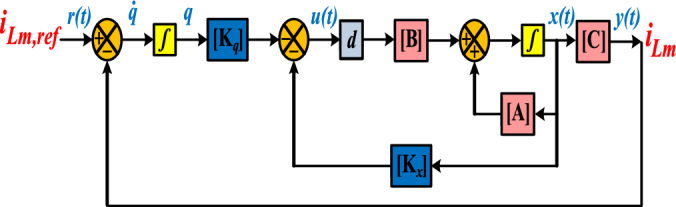
Block diagram of the pole-placement control technique.

**Fig. 11:**
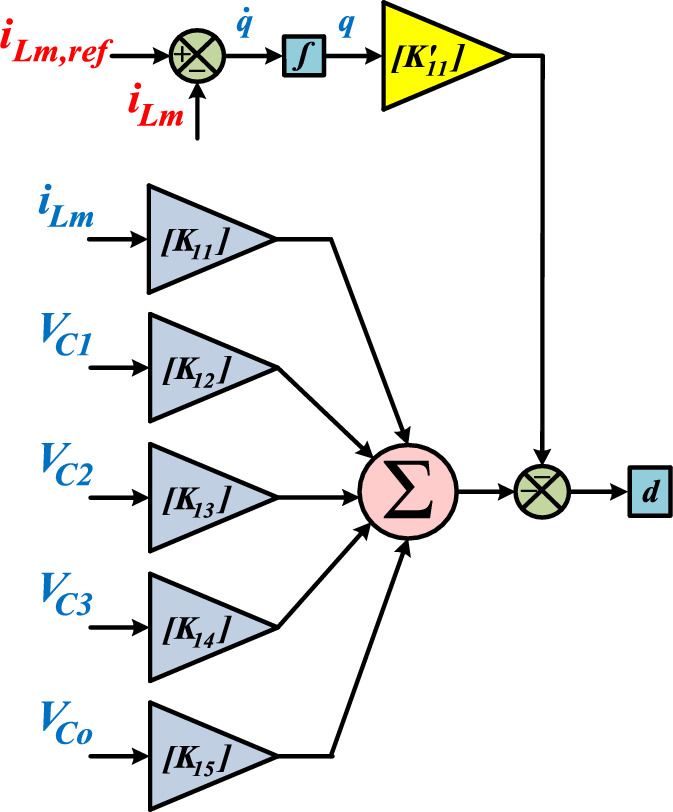
Current regulator loop of the coupled inductor.

To ensure suitable Gain Margin and Phase Margin values (GM ≥ 10 and 60 ≤ PM ≤ 80), the trial and error method is employed to determine the positions of the closed-loop poles. By following this approach, the bode plot of the control system for the proffered circuit is demonstrated in Fig. 9. As demonstrated in Fig. 9, the gain margin values for the inductor L_m_ exceed 10 (GM (i_Lm_) > 10), and the phase margin for the control path (closed-loop) of i_Lm_ is 73.6953, which is deemed acceptable. Additionally, Figs. 10 and 11 depict the block diagram of the pole-placement control method and the current regulator loop of the coupled inductor, respectively.

## Comparison study

In order to showcase the effectiveness of the proffered circuit, an examination is conducted to compare its performance with that of other configurations. Table 1 outlines the characteristics of the suggested converter alongside alternative configurations, covering aspects such as voltage gain, maximum stress on power switches and diodes, capability for soft switching, component count, nominal power, efficiency, and ability for common ground utilization.

**Table 1:** Comparison between the propounded configuration and other circuits.

References	Voltage gain	Maximum voltage stress on switches	Maximum voltage stress on diodes	Soft switching capability	Number of *					T**/Power/Efficiency/Common ground
					S	D	C	L	CI	
Pro	$\frac{1 + 2n - D - nD}{{1 - 2D}}$	$\frac{{(1 - D)V_{O} }}{1 + 2n - D - nD}$	$\frac{{(n - D + 1)V_{O} }}{1 + 2n - D - nD}$	Yes	2	5	4	0	1	12/150W/95.47%/Yes
^15^	$\frac{2n}{{1 - 2D}}$	$\frac{1}{2n}V_{O}$	$\frac{1}{2}V_{O}$	No	2	5	7	1	1	16/240W/92%/No
^16^	$\frac{2 + n - D}{{1 - 2D}}$	$\frac{{V_{O} }}{2 + n - D}$	$\frac{{(1 + n)V_{O} }}{2 + n - D}$	Yes	2	3	4	1	1	11/300W/96.8%/Yes
^17^	$\frac{3 - 2D}{{1 - 2D}}$	$\frac{{V_{O} }}{3 - 2D}$	$\frac{{V_{O} }}{3 - 2D}$	No	1	4	6	3	0	14/400W/91.25%/No
^18^	$\frac{1 + D}{{1 - 2D}}$	$\frac{{V_{O} }}{1 + D}$	$\frac{{V_{O} }}{1 + D}$	No	1	3	4	2	0	10/100W/92.5%/No
^19^	$\frac{3 - 2D}{{1 - 2D}}$	$\frac{{V_{O} }}{3 - 2D}$	$\frac{{V_{O} }}{3 - 2D}$	No	1	4	5	2	0	12/150W/86%/Yes
^20^	$\frac{{3 - 3D - 2D^{2} }}{(1 - 2D)(1 - D)}$	$\frac{{(1 - D)V_{O} }}{{3 - 3D - 2D^{2} }}$	$\frac{{(2 - 3D)V_{O} }}{{3 - 3D - 2D^{2} }}$	No	2	4	5	3	0	14/200W/93.8%/No
^21^	$\frac{3 - 2D}{{1 - 2D}}$	$\frac{{V_{O} }}{3 - 2D}$	$\frac{{V_{O} }}{3 - 2D}$	No	1	5	6	2	0	14/130W/92%/Yes
^22^	$\frac{2}{1 - 2D}$	$\frac{{V_{O} }}{2}$	$V_{O}$	No	2	4	6	4	0	16/200W/93%/No
^23^	$\frac{3 - D}{{(1 - D)^{2} }}$	$\frac{{DV_{O} }}{{(1 - D)^{2} }}$	$\frac{{V_{O} }}{1 - D}$	No	1	6	6	3	0	16/100W/91%/Yes
^24^	$\frac{{1 + n + 2nD - 2nD^{2} }}{{(1 - D)^{2} }}$	$\frac{{V_{O} }}{{1 + n + 2nD - 2nD^{2} }}$	$\frac{{(1 + n)V_{O} }}{{1 + n + 2nD - 2nD^{2} }}$	No	1	6	4	1	1	13/1014W/95.94%/Yes
^25^	$\frac{2 - D}{{1 - 2D}}$	$\frac{{V_{O} }}{2 - D}$	$\frac{{V_{O} }}{2 - D}$	No	1	3	4	2	0	10/100W/92%/Yes
^26^	$\frac{2n + 1 + D}{{(1 - D)^{2} }}$	$\frac{{(1 + D)V_{O} }}{2n + 1 + D}$	$\frac{{2nV_{O} }}{2n + 1 + D}$	Yes	2	5	5	1	1	14/280W/95.05%/Yes
^27^	$\frac{1 + n(1 - D)}{{1 - 2D}}$	$\frac{{V_{O} }}{1 + n(1 - D)}$	$\frac{{nV_{O} }}{1 + n(1 - D)}$	Yes	2	2	4	1	1	10/100W/95.6%/Yes
^28^ BFSL	$\frac{2 - D}{{(1 - 2D)(1 - D)}}$	$\frac{{(2 - D)V_{O} }}{(1 - D)}$	$\frac{{(2 - D)V_{O} }}{(1 - D)}$	No	1	5	5	3	0	14/200W/91.4%/Yes
^28^ BCL	$\frac{2}{1 - 2D}$	$2V_{O}$	$2V_{O}$	No	1	4	4	3	0	12/200W/92.2%/Yes
^29^	$\frac{3 - 2D}{{1 - 2D}}$	$\frac{{V_{O} }}{3 - 2D}$	$\frac{{V_{O} }}{3 - 2D}$	No	1	4	5	2	0	12/150W/86%/Yes

*Number of Switches/Diodes/Capacitors/Inductors/Coupled Inductors.

**Total number of components.

**Fig. 12:**
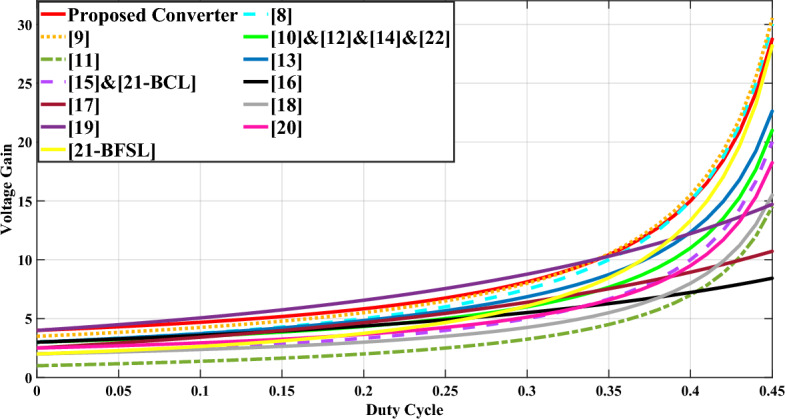
Voltage gain variations per different duty cycles for the compared step-up circuits with n = 1.5.

Table 1 presents a comprehensive overview of the converters discussed in references^15–19^. The focus of this paper revolves around the UHSU circuit, with voltage gain serving as the primary parameter for comparison. Figure 12 illustrates the variations in voltage gain across different duty cycles for the compared step-up configurations, with the depicted curves derived from the data expressed in Table 1. The results indicate that the suggested circuit outperforms other structures, particularly surpassing the UHSU configurations in references^23,24,26^. However, it's worth noting that the structures in references^15,16^ achieve a higher voltage gain per D > 0.4, albeit marginally higher than that of the proposed circuit. Nevertheless, the proposed converter demonstrates superiority across most factors examined in Table 1.

**Fig. 13:**
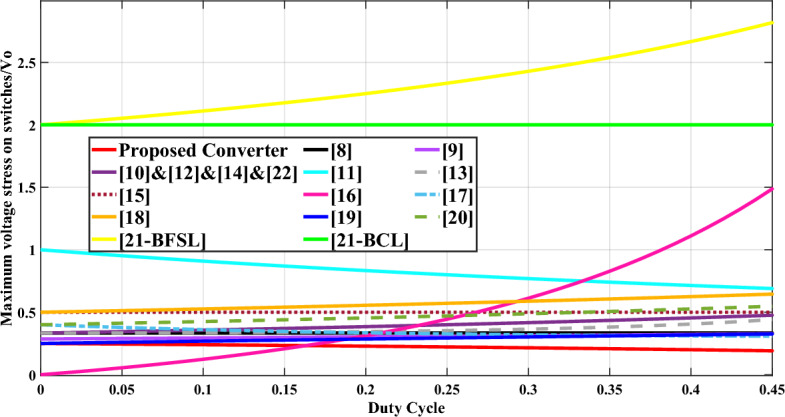
Comparison of the maximum voltage stress on switches versus duty cycle (n = 1.5).

In Table 1, the second column presents the maximum voltage rating of the power switches. The variation of this parameter across different duty cycles is illustrated in Fig. 13. The graph in Fig. 13 highlights that the suggested circuit exhibits superior performance in terms of the maximum voltage stress experienced by the switches compared to other configurations. Notably, the voltage across the power switches is considerably low in the suggested circuit, enabling the selection of a smaller inductor for the propounded design.

**Fig. 14:**
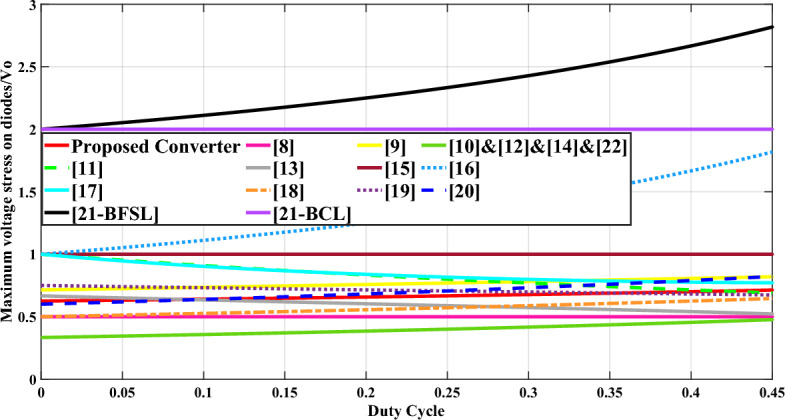
Comparison of the maximum voltage stress on diodes versus duty cycle (n = 1.5).

The third column in Table 1 provides information on the maximum voltage stress experienced by the diodes. Figure 14 displays the curve representing the maximum voltage stress on diodes across various duty cycles, considering a turns ratio (n) of 1.5. It is evident from Fig. 14 that the maximum voltage stress observed in the suggested configuration is lower compared to configurations presented in references^16,18,22–24,27,28^.

The fourth column of Table 1 indicates the presence of soft switching capability on semiconductor components. The proposed converter demonstrates ZCS turn-on, as illustrated in Fig. 5, which contributes to enhancing its efficiency. In contrast, configurations outlined in references^15,17–25,28,29^ lack soft switching capability on their semiconductor components.

The fifth column of Table 1 presents the count of switches, diodes, capacitors, inductors, and CIs. Unlike other configurations that typically employ at least two cores, the proposed converter utilizes only one core. This design choice enhances power density and reduces size due to the streamlined use of a single core. The total number of components is also noted in the subsequent column. In the suggested circuit, this count equals or is lower than most UHSU and high step-up circuits (equaling or being less than structures in^15,17,19–24,26,28,29^). However, it's important to note that the number of components alone may not fully capture the cost and power density metrics, as some converters with a higher component count may have lower costs and higher power densities due to the lower voltage and current ratings of the components. A cost analysis comparing the proposed converter with other structures is outlined in Table 2. Here, it's evident that the cost of the suggested converter is the most economical. The estimated costs of components were sourced from listings on platforms like AMAZON and EBAY, summarized succinctly in Table 2. The determination of the proffered circuit's volume and size distribution is detailed in Table 3. Notably, the CI constitutes a significant portion of the overall volume, followed closely by the capacitors, with the high-voltage side capacitor being the dominant contributor. In contrast, semiconductor devices make a comparatively minor contribution to the overall volume. Additionally, for comparative purposes, the power density is also provided in Table 4. The proposed converter achieves a theoretical power density of 150 W per 40,292.24 mm^3^, surpassing that of other circuits. It's worth noting that the cost and power density metrics were calculated for converters sharing component types. In the final column of Table 1, the power, efficiency, and common ground capability of the configurations are discussed. The propounded circuit is constructed for 150 W with an impressive efficiency of 95.47%, outperforming most other structures. Additionally, common ground capability between the input and output is highlighted as crucial, as configurations lacking this capability may be susceptible to electromagnetic interference noises affecting the circuits. Unlike converters found in^15,17,18,20,22^, the proffered structure possesses common ground capability. Comparative analysis reveals the superior voltage gain of the proposed configuration, along with reduced voltage stress on its power switches and diodes, all achieved at a low cost and with high power density. Moreover, owing to the low voltage stress on the power switch, the presented circuit necessitates a smaller inductor.

**Table 2:** Cost comparison between the proffered configuration and other circuits.

References	Cost of switches	Cost of Diodes	Cost of Capacitors	Cost of Cores	Total Cost
Pro	2 × 0.363$	2 × 0.476$ 3 × 0.375$	1 × 0.3145$, 2 × 0.4495$ 1 × 0.799$	1 × 2.7325$	7.548$
^15^	2 × 1.235$	1 × 0.77$ 4 × 0.689$	1 × 0.988$, 2 × 0.599$ 1 × 1.141$, 1 × 1.415$ 2 × 0.799$	2 × 5.05$	22.436$
^17^	1 × 5.942$	4 × 1.35$	2 × 14.43$, 1 × 7.27$#3 × 6.70$	3 × 4.22$	80.232$
^18^	The total cost, as calculated by the authors in^18^, is equal to: 7.95$				
^29^	1 × 1.18$	4 × 0.99$	5 × 0.988$	2 × 1.83$	13.74$

**Table 3:** Power density of the propounded circuit.

Components	Specification	Volume
Switches	IRF2807PbF Length:23.74 mm Width:10.67 mm Thickness:4.83 mm	2 × 1223.46 = 2446.92 mm^3^
Diodes	BYV32E-200 Length:31 mm Width:10.3 mm Thickness:4.7 mm SBR10U300CT Length:29.15 mm Width:10.31 mm Thickness:4.67 mm	(2 × 1500.71) + (3 × 1403.5) = 7211.92 mm^3^
Capacitors	C_1_ Diameter:6.3 mm Height:11 mm C_2_, C_3_ Diameter:13 Height:20 C_O_ Diameter:16 Height:25	(342.90) + (3 × 2654.65) + (5026.55) = 13,333.4 mm^3^
Coupled Inductor	E42/21/15 core	17300 mm^3^
	Total volume:	40,292.24 mm^3^
	Power Density:	3.72 mW/mm^3^

**Table 4:** Power density comparison between the proffered circuit and other configurations.

References	S.V* (mm^3^)	D.V* (mm^3^)	C.V* (mm^3^)	I.V* (mm^3^)	T.V* (mm^3^)	P.D* (mm^3^)
Pro	2446.92	7211.92	13,333.4	17,300	40,292.24	150W/40,292.24 = 3.72 mW/mm^3^
^15^	6174.88	9769.54	46,806.57	20,880	83,630.99	240W/83,630.99 = 2.86 mW/mm^3^
^17^	3480.82	15,637.60	259,392	106,800	385,310.42	400W/385,310.42 = 1.03 mW/mm^3^
^29^	2984.84	5955.44	47,516.6	23,000	79,456.88	150W/79,456.88 = 1.88 mW/mm^3^

*S.V Switches Volume, *D.V Diodes volume, *C.V Capacitors Volume, *I.V Inductors Volume, *T.V Total Volume, *P.D Power Density.

## Experimental results

**Fig. 15:**
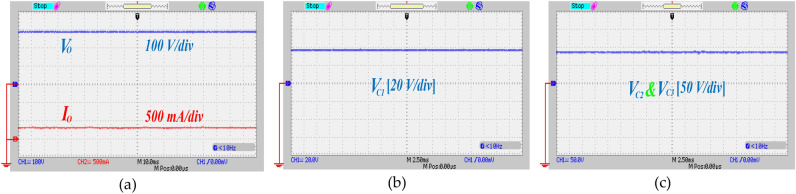
The experimental waveforms of output port and capacitors, (**a**) voltage and current of the output port, (**b**) voltage across the capacitor C_1_, (**c**) voltage across the capacitors C_2_ and C_3_.

**Fig. 16:**
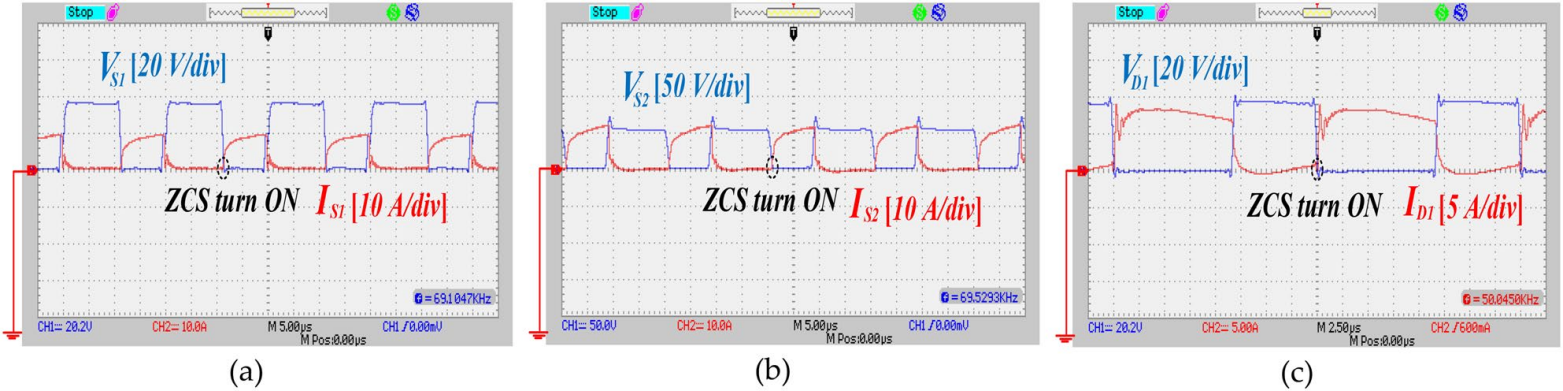
The experimental waveforms of power switches and diode D_1_, (**a**) voltage and current of the power switch S_1_, (**b**) voltage and current of the power switch S_2_, (**c**) voltage and current of the diode D_1_.

**Fig. 17:**
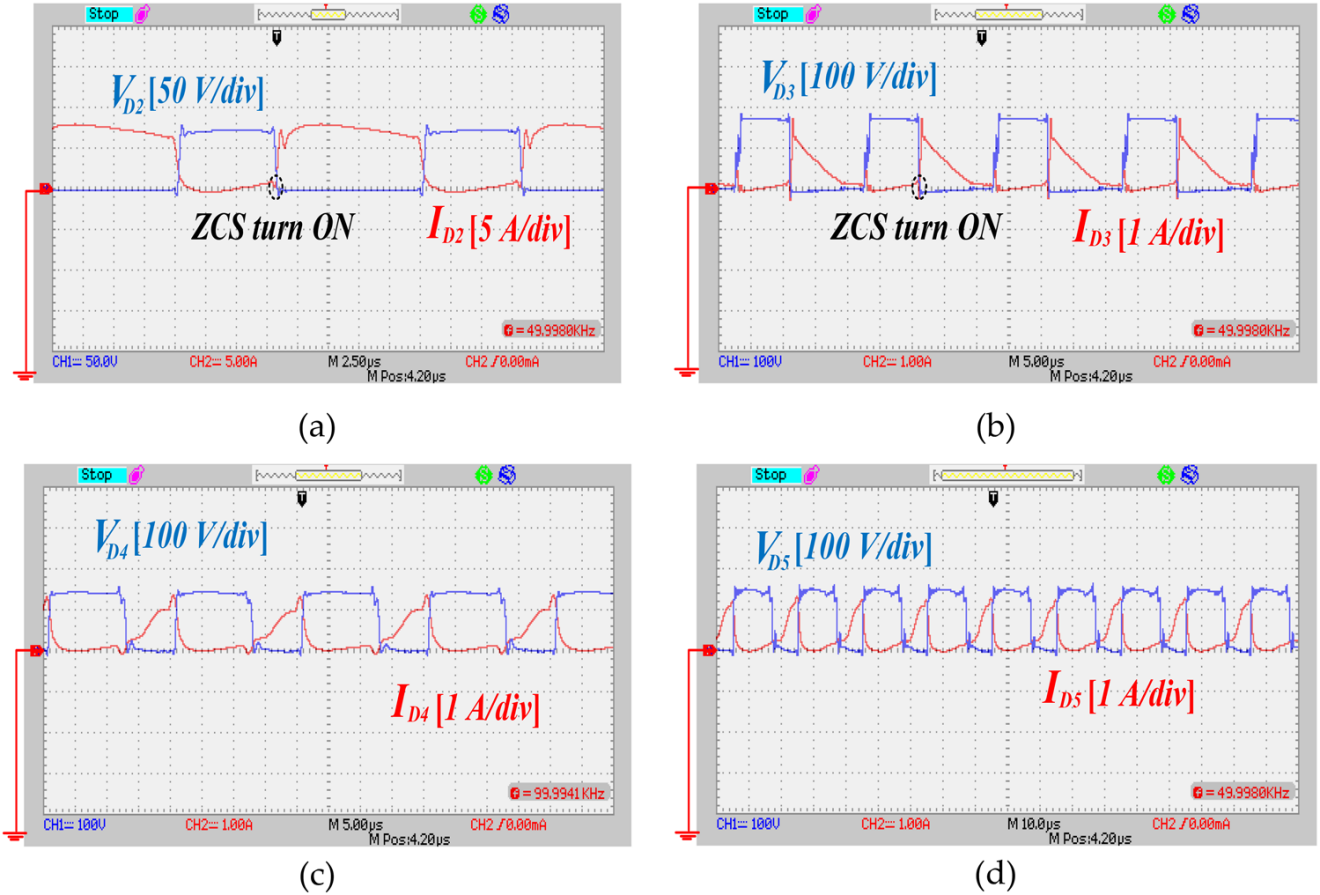
The experimental waveforms of diodes, (**a**) voltage and current of the diode D_2_, (**b**) voltage and current of the diode D_3_, (**c**) voltage and current of the diode D_4_, (**d**) voltage and current of the diode D_5_.

To validate the theoretical analysis and actual execution of the proffered circuit, an experimental prototype with a power rating of 150 W is constructed. Table 5 outlines the key specifications of the propounded UHSU converter. Figure 15a depicts the measured output voltage and current, which are recorded at 290 V and 0.32 A, respectively. The theoretical output voltage predicted by Eq. (14) is approximately 300 V, closely matching the experimental result. This alignment validates the operation of the proffered circuit. Figure 15b,c display the voltage across capacitors C_1_, C_2_, and C_3_, measured at 38 V and 87 V, respectively. Theoretical calculations based on Eqs. (12) and (13) predict capacitor voltages of 40 V for C_1_ and 90 V for C_2_ and C_3_. Figure 16a presents the voltage across power switch S_1_ and the corresponding current through S_1_, which were measured at 37 V and 10 A, respectively. Additionally, ZCS on switch S_1_ is depicted in the same figure. In Fig. 16b, the voltage and current profiles of switch S_2_ are demonstrated, showing a voltage across S_2_ of 55 V and a current through S_2_ of 11 A. ZCS condition on switch S_2_ is also clearly visible in the same figure. Diode D_1_ enters conduction under ZCS condition, as shown in Fig. 16c. Voltage and current measurements across diode D_1_ indicate values of 38 V and 8 A, respectively. Similarly, diode D_2_ displays ZCS condition, with readings of 60 V and 8 A, as depicted in Fig. 17a. Under ZCS condition, diode D_3_ exhibits forward bias, registering a voltage of 180 V and a current of 1.7 A, as seen in Fig. 17b. The voltage and current profiles of diodes D_4_ and D_5_ mirror each other, as shown in Fig. 17c,d respectively, with measurements indicating 140 V and 1.4 A. Overall, Figs. 15, 16 and 17 collectively demonstrate that the experimental outcomes of the proffered converter closely correspond with the theoretical predictions derived from analysis, thus validating the converter's performance.

**Table 5:** Component characteristic of the implemented circuit.

Parameters	Values
Rated power (*P*_*o*_)	150 *W*
Input voltage	20 V
Output voltage	300 V
Switching Frequency (*f*_*s*_)	50 kHz
Turns Ratio *n* (*N*_*S*_*/N*_*P*_)	1.5
Magnetizing Inductor (*L*_*m*_)	500 µH
Leakage Inductor (*L*_*k*_)	3 µH
Power Switches	IRF2807PbF
Diodes (*D*_*1*_*,D*_*2*_)	BYV32E-200
Diodes (*D*_*3*_*,D*_*4*_*,D*_*5*_)	SBR10U300CT
Core type	E42/21/15
Capacitor (*C*_*1*_)	22 µF/100 V
Capacitors (*C*_*2*_,* C*_*3*_)	22 µF/250 V
Capacitor (*C*_***o***_)	47 µF/450 V

**Fig. 18:**
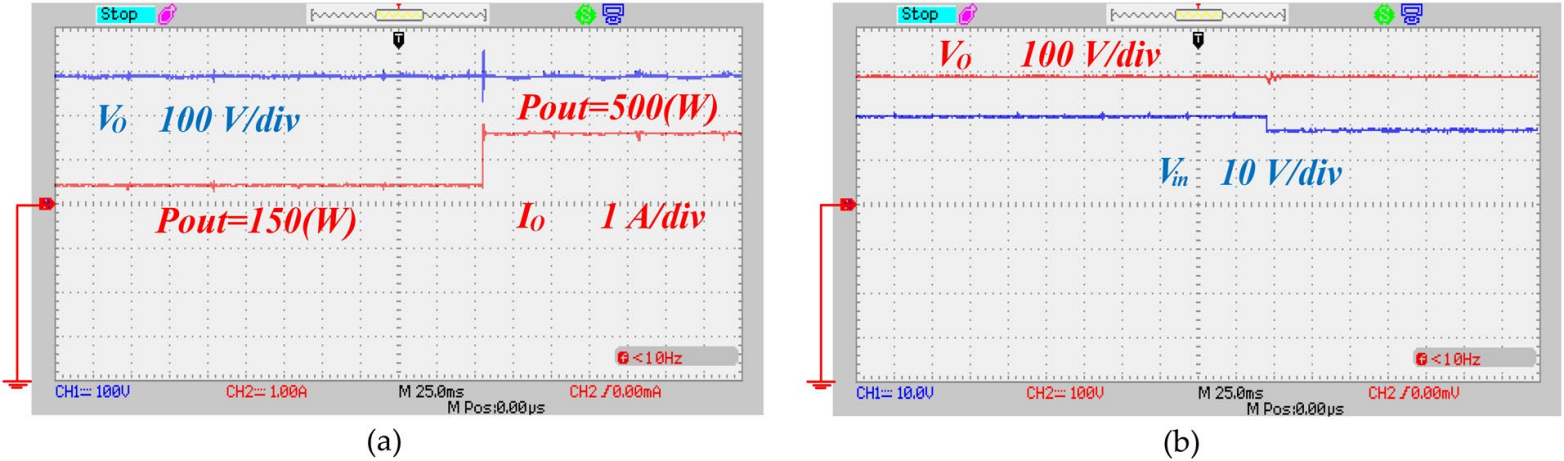
Dynamic response of the proposed converter, (**a**) step change of the load, (**b**) step change of the input voltage.

The proposed converter was tested under various load conditions and input values. In Fig. 18a, the output voltage of the converter initially registers around 290 V with a power output of about 150 W. When the load is suddenly altered and the output power is adjusted to 500 W, the output voltage remains relatively stable after brief transient fluctuations. The output voltage deviates only slightly from the reference value, demonstrating the stability of the closed-loop system in maintaining the output voltage close to the target. Figure 18b depicts the output voltage response when the input voltage suddenly drops from 20 to 16 V. It is evident from Fig. 18b that the output voltage shows minimal variation in response to the input change.

## Conclusions

This paper introduces a non-isolated UHSU DC–DC circuit employing the CI method. The proffered UHSU configuration achieves an elevated voltage gain by increasing the turn ratio of the CI. Key merits of the proffered configuration include a high voltage gain with high efficiency, zero current switching of power switches and diodes during the ON-state, minimal voltage stress across semiconductor components, and the flexibility provided by two control parameters for adjusting the circuit's voltage gain (duty cycle and turns-ratio of the CIs). Additionally, the configuration is capable of generating high output voltage with a reduced duty cycle, thereby minimizing conduction losses in the switches. Furthermore, the proposed design offers high power density and cost-effectiveness. The suggested UHSU configuration offers a practical answer for converting low DC to high DC in DC microgrid application.

## Data Availability

All data generated or analyzed during this study are included in this published article.
